# Risk factors for future exacerbations in patients with COPD treated with dual LABA/LAMA

**DOI:** 10.1080/07853890.2026.2722722

**Published:** 2026-09-15

**Authors:** Qing Song, Tingting Chen, Dan Peng, Chengrui Liu, Rensen Xiao, Qinqin Yao, Xiaoling Guo, Longhua Sun, Wenxin Yuan, Fang Huang

**Affiliations:** ^a^ Postdoctoral Innovation Practice Base, The First Affiliated Hospital, Jiangxi Medical College, Nanchang University, Nanchang, People’s Republic of China; ^b^ Department of Pulmonary and Critical Care Medicine, the First Affiliated Hospital of Nanchang University, Nanchang, China; ^c^ Department of Pulmonary and Critical Care Medicine, Jiangxi Hospital of China-Japan Friendship Hospital, Nanchang, China; ^d^ Department of General Medicine, The Fourth People’s Hospital of Nanchang County, Nanchang, Jiangxi, China; ^e^ Ultrasound Department, the First Affiliated Hospital of Nanchang University, Nanchang, China

**Keywords:** Chronic obstructive pulmonary disease, Laba/LAMA, exacerbation, GOLD grades

## Abstract

**Background:**

The Global Initiative for Chronic Obstructive Lung Disease (GOLD) 2026 report revised the combined chronic obstructive pulmonary disease (COPD) assessment. Patients in groups B and E are recommended dual long-acting β_2_-agonist (LABA)/long-acting muscarinic antagonist (LAMA) as the initial inhalation therapy. However, some patients still experience exacerbations during treatment. This study aimed to investigate the risk factors for future exacerbations in patients with COPD treated with dual LABA/LAMA.

**Methods:**

This was a retrospective cohort study. Baseline data included demographic characteristics, pulmonary function, symptom scores, GOLD grades, GOLD groups, the number of exacerbations and hospitalizations in the past year, LABA/LAMA, and comorbidities were collected. The number of future exacerbations was collected during one year of follow-up.

**Results:**

A total of 1076 patients with COPD were included. There were 284 (26.4%) patients experienced exacerbations during follow-up. Patients who experienced exacerbations were older and had higher clinical COPD questionnaire scores, a great number of exacerbations and hospitalizations in the past year, and a higher proportion of GOLD grades 3–4. Logistic regression analysis showed that GOLD grades 3–4 (odds ratio [OR] = 1.343, 95% confidence intervals [CI] = 1.016–1.776), one exacerbation in the past year (OR = 1.763, 95% CI = 1.248–2.489), and ≥ 2 exacerbations in the past year (OR = 2.514, 95% CI = 1.792–3.525) were independent risk factors for experiencing exacerbations. Furthermore, in group B, logistic regression analysis showed that GOLD grades 3–4 (OR = 1.808, 95% CI = 1.096–2.981) and hypertension (OR = 3.400, 95% CI = 1.712–6.752) were independent risk factors for experiencing exacerbations. In group E, logistic regression analysis showed that ≥ 2 exacerbations in the past year (OR = 1.426, 95% CI = 1.021–1.993) was an independent risk factor for experiencing exacerbations.

**Conclusions:**

Patients with COPD treated with LABA/LAMA remained at high risk of future exacerbations. GOLD grades 3–4 and exacerbations in the past year were associated with future exacerbations and may warrant particular attention from clinicians.

## Introduction

Chronic obstructive pulmonary disease (COPD) is a prevalent, preventable, and treatable chronic respiratory disease characterized by persistent respiratory symptoms and airflow limitation, imposing a substantial disease burden worldwide [1,2]. Exacerbations of COPD are critical events that lead to rapid deterioration of respiratory symptoms, accelerated decline in pulmonary function, increased risk of hospitalization and mortality, and elevated medical costs [3–5]. Therefore, reducing the risk of future exacerbations has become one of the core goals in the long-term management of COPD.

The Global Initiative for Chronic Obstructive Lung Disease (GOLD) 2026 report updated the comprehensive assessment strategy for COPD and refined the patient grouping criteria. For patients with COPD in groups B and E, dual long-acting β_2_-agonist/long-acting muscarinic antagonist (LABA/LAMA) inhalation therapy is recommended as the initial treatment option [6]. Dual LABA/LAMA therapy can effectively relieve clinical symptoms, improve exercise tolerance and quality of life, and has been widely used in clinical practice [7–9].

However, the overall COPD population is highly heterogeneous, with different therapeutic strategies across patient subgroups, making the clinical translation of predictive models challenging. LAMA monotherapy is mainly prescribed for mildly symptomatic patients with a very low risk of exacerbation. By contrast, LABA/LAMA/inhaled corticosteroid (ICS) therapy is indicated for frequent exacerbators with elevated eosinophil counts or asthma–COPD overlap, who have the highest risk of recurrent severe exacerbations and pneumonia-related adverse events. Therefore, patients receiving LABA/LAMA represent the largest intermediate-risk transitional population. This subgroup has a considerable residual risk of moderate-to-severe exacerbations that cannot be fully eliminated by dual bronchodilation alone, yet lacks validated, dedicated risk prediction tools [10]. Their risk factors and exacerbation triggers differ substantially from those of patients receiving the other two treatment regimens, supporting independent subgroup analysis. In addition, existing prediction models have important limitations in the LABA/LAMA subgroup. Most previously published COPD exacerbation prediction models were developed using mixed overall COPD populations or patients receiving triple therapy with a heavy exacerbation burden. Identifying high-risk individuals receiving dual LABA/LAMA therapy may enable clinicians to intensify monitoring, optimize follow-up frequency, and promptly step up to triple therapy before severe exacerbations occur, thereby addressing this important research gap.

Accordingly, this retrospective cohort analysis was performed to investigate the risk factors for future exacerbations in patients with COPD receiving dual LABA/LAMA therapy, with the aim of providing evidence to improve the prognosis of patients with COPD.

## Patients and methods

### Study participants

This retrospective, real-world cohort study included patients registered at a large tertiary hospital in China between 1 December 2020 and 31 January 2025. Patients had been diagnosed with COPD according to the GOLD 2021 report [6]: the ratio of forced expiratory volume in 1 s to forced vital capacity (FEV1/FVC) was <0.70 after bronchodilator inhalation. Patients with active tuberculosis, asthma, or severe heart, liver, or kidney disease were excluded. According to the GOLD 2026 report, triple inhaled therapy (LABA/LAMA/ICS) is recommended for patients with blood eosinophil counts ≥300 cells/μL; therefore, these patients were also excluded from our cohort.

The exemption from obtaining informed consent from the participants has been obtained due to the nature of the research.

### Data collection

At the patients’ first hospital visit, baseline data were collected, including age, sex, education level, body mass index, smoking history, smoking exposure (pack-years), biofuel exposure, FEV1% predicted (FEV1%pred), FEV1/FVC, COPD Assessment Test (CAT) score, modified Medical Research Council (mMRC) dyspnea score, Clinical COPD Questionnaire (CCQ) score, GOLD grades, GOLD groups, the number of exacerbations and hospitalizations in the past year, comorbidities (including chronic heart disease, hypertension, lung cancer, diabetes, and bronchiectasis), and inhaled treatment regimen, including LABA/LAMA.

A dedicated specialist physician instructed patients on inhaler technique in a separate consultation room. Thus, all patients received standardized inhaler technique education from a professor when inhaled therapy was initiated.

All patients completed one year of follow-up, during which the number of exacerbations was collected by telephone interview. Patients were interviewed to determine whether they had sought care for pulmonary exacerbations at community clinics or other hospitals, and to verify ongoing adherence to inhaled medications. Patients whose inhaled treatment regimen was adjusted during follow-up were excluded from the study. In addition, all patients presenting with respiratory exacerbations during follow-up underwent standardized SARS-CoV-2 testing. Respiratory events confirmed to be caused by COVID-19 infection were identified and excluded from the count of COPD exacerbations.

### Study procedure

In this study, patients with COPD in groups B and E who were treated with LABA/LAMA were enrolled at their first hospital visit. The patients were divided into an exacerbation group and a non-exacerbation group according to whether an exacerbation occurred during the follow-up period.

### Variable definition

Group E was defined as patients who had experienced one or more moderate or severe exacerbations in the past year. Group B was defined as patients who had not experienced an exacerbation in the past year, and had a CAT score of ≥ 10, or a mMRC score of ≥ 2 [6]. An exacerbation (including future exacerbations) was defined as a respiratory event accompanied by worsening respiratory symptoms requiring COPD-related treatment, including antibiotics, or oral corticosteroids, or hospitalization (moderate and severe exacerbations) [11]. GOLD grades were based on the post-bronchodilator FEV1%pred as follows: GOLD grades 1–2, FEV1 ≥ 50% pred and GOLD grades 3–4, FEV1 < 50% pred [6]. Biofuel exposure was defined as the use of biomass fuels for cooking or heating for at least two hours per day for at least one year [12]. A former smoker was defined as a person with a smoking history of ≥10 pack-years who had quit smoking for more than 6 months, whereas a current smoker was defined as a person with a smoking history of ≥10 pack-years [13].

### Statistical analysis

SPSS version 26.0 (IBM Corp., Armonk, NY, USA) and Free Statistics software version 2.2.0 (Beijing, China) were used for the statistical analyses. Student’ t test was used to analyze continuous variables with a normal distribution and homogeneity of variance, and the data are presented as mean ± standard deviation. Otherwise, continuous variables were analyzed using nonparametric tests and are presented as median and interquartile range. The chi-square test was used to analyze categorical variables. Multivariate logistic regression was used to calculate odds ratio (OR) and 95% confidence intervals (CI). For multivariate logistic regression analysis of the overall cohort, variables with *p* < 0.05 in Table 3 were entered into the model. For subgroup multivariate analyses of groups B and E, only variables with *p* < 0.05 in the corresponding univariate analyses were included in each multivariate model. There was no multicollinearity among variables and the results were showed in the Supplement Tables 1 and 2. A *p* value of <0.05 was considered statistically significant.

**Table 1.** The clinical characteristics of the patients.

Variables	Total (*n* = 1076)	Group B (*n* = 442)	Group E (*n* = 634)	*p*-values
Age (years), (Mean ± SD)	66.3 ± 8.2	65.8 ± 7.8	66.7 ± 8.4	0.062
Sex, n (%)				**0.043**
Male	948 (88.1)	400 (90.5)	548 (86.4)	
Female	128 (11.9)	42 (9.5)	86 (13.6)	
Education level, n (%)				0.082
Under junior high school	834 (77.5)	331 (74.9)	503 (79.3)	
Over high school	242 (22.5)	111 (25.1)	131 (20.7)	
BMI (kg/m^2^), (Mean ± SD)	22.7 ± 3.5	23.0 ± 3.6	22.5 ± 3.5	**0.018**
Smoke history, n (%)				**0.035**
Never smoker	202 (18.8)	75 (170)	127 (20.0)	
Former smoker	360 (33.5)	135 (30.5)	225 (35.5)	
Current smoker	514 (47.7)	232 (52.5)	282 (44.5)	
Smoking, (pack/year) (Median, IQR)	40.0 (20.0, 54.0)	40.0 (22.5, 53.0)	40.0 (20.0, 55.0)	0.798
Biofuel exposure, n (%)				0.900
Yes	387 (36.0)	158 (35.7)	229 (36.1)	
No	689 (64.0)	284 (64.3)	405 (63.9)	
FeNO (ppb) (Median, IQR)	16.0 (11.0, 21.0)	17.0 (11.0, 22.0)	16.0 (11.0, 21.0)	0.384
Eosinophil count (×10^9^) (Median, IQR)	0.1 (0.1, 0.2)	0.1 (0.1, 0.2)	0.1 (0.1, 0.2)	0.663
Pulmonary function, (Mean ± SD)				
FEV1 %pred	56.1 ± 20.0	57.6 ± 19.9	55.0 ± 20.0	**0.039**
FEV1/FVC	49.3 ± 11.9	49.7 ± 11.4	49.0 ± 12.2	0.368
CAT scores, (Mean ± SD)	14.6 ± 5.8	14.1 ± 5.0	14.9 ± 6.2	**0.017**
CAT scores, n (%)				**0.005**
<10	183 (17.0)	58 (13.1)	125 (19.7)	
≥20	893 (83.0)	384 (86.9)	509 (80.3)	
mMRC scores, (Median, IQR)	2 (1, 2)	2 (1, 2)	2 (1, 3)	0.100
mMRC scores, n (%)				0.834
0–1	337 (31.3)	140 (31.7)	197 (31.1)	
≥2	739 (68.7)	302 (68.3)	437 (68.9)	
CCQ scores, (Mean ± SD)	21.5 ± 6.7	20.1 ± 6.0	22.5 ± 7.0	**<0.001**
GOLD grades, n (%)				0.055
1–2	625 (58.1)	272 (61.5)	353 (55.7)	
3–4	451 (41.9)	170 (38.5)	281 (44.3)	
Comorbidities, n (%)				
Chronic heart disease	62 (5.8)	23 (5.2)	39 (6.2)	0.512
Hypertension	95 (8.8)	43 (9.7)	52 (8.2)	0.385
Lung cancer	42 (3.9)	17 (3.8)	25 (3.9)	0.936
Diabetes	19 (1.8)	7 (16)	12 (1.9)	0.705
Bronchiectasis	96 (8.9)	22 (5.0)	74 (11.7)	**<0.001**
Exacerbations in the past year, (Median, IQR)	1 (0, 2)	0 (0, 0)	1 (1, 3)	**<0.001**
Exacerbations in the past years, n (%)				**<0.001**
0	442 (41.1)	442 (100.0)	0 (0)	
1	324 (30.1)	0 (0)	324 (51.1)	
≥2	310 (28.8)	0 (0)	310 (48.9)	
Hospitalizations in the past year, (Median, IQR)	0 (0, 1)	0 (0, 0)	1 (0, 1)	**<0.001**
Hospitalizations in the past year, n (%)				**<0.001**
0	680 (63.2)	442 (100.0)	238 (37.5)	
≥1	396 (36.8)	0 (0)	396 (62.5)	

Notes. The bold *p*-values indicate statistical significance. BMI: Body Mass Index; CCQ: Clinical COPD Questionnaire; COPD: Chronic Obstructive Pulmonary Disease; CAT: COPD Assessment Test; FEV1%pred: Forced Expiratory Volume in one second percentage predicted; FEV1: Forced Expiratory Volume in one second; FVC: Forced Vital Capacity; GOLD: Global Initiative for Chronic Obstructive Lung Disease; IQR: InterQuartile Range; mMRC: modified Medical Research Council.

**Table 2.** The future exacerbation of the patients durl,kjing one year of follow-up.

Variables	Total (*n* = 1076)	Group B (*n* = 442)	Group E (*n* = 634)	*p*-values
Exacerbations during one year, (Median, IQR)	0 (0, 1)	0 (0, 0)	0 (0, 1)	**<0.001**
Exacerbations, n (%)				**<0.001**
Yes	284 (26.4)	80 (18.1)	204 (32.2)	
No	792 (73.6)	362 (81.9)	430 (67.8)	

Notes. The bold *p*-values indicate statistical significance. IQR: InterQuartile Range.

**Table 3.** The clinical characteristics of the total patients experienced exacerbation during follow-up.

Variables	Non-exacerbation (*n* = 792)	Exacerbation (*n* = 284)	*p*-values
Age (years), (Mean ± SD)	66.0 ± 8.1	67.2 ± 8.1	**0.044**
Sex, n (%)			0.492
Male	701 (88.5)	247 (87.0)	
Female	91 (11.5)	37 (13.0)	
Education level, n (%)			0.725
Under junior high school	616 (77.8)	218 (76.8)	
Over high school	176 (22.2)	66 (23.2)	
BMI (kg/m^2^), (Mean ± SD)	22.8 ± 3.5	22.4 ± 3.6	0.196
Smoke history, n (%)			0.050
Never smoker	142 (17.9)	60 (21.1)	
Former smoker	254 (32.1)	106 (37.3)	
Current smoker	396 (50.0)	118 (41.5)	
Smoking, (pack/year) (Median, IQR)	40.0 (20.0, 54.0)	40.5 (20.0, 55.0)	0.684
Biofuel exposure, n (%)			0.902
Yes	284 (35.9)	103 (36.3)	
No	508 (64.1)	181 (63.7)	
FeNO (ppb) (Median, IQR)	16.0 (11.0, 22.0)	16.0 (11.0, 20.0)	0.179
Eosinophil count (×10^9^) (Median, IQR)	0.1 (0.1, 0.2)	0.1 (0.1, 0.2)	0.683
Pulmonary function, (Mean ± SD)			
FEV1 %pred	56.5 ± 19.8	55.0 ± 20.3	0.286
FEV1/FVC	49.6 ± 11.9	48.5 ± 11.8	0.219
CAT scores, (Mean ± SD)	14.4 ± 5.6	15.0 ± 6.1	0.118
CAT scores, n (%)			0.496
<10	131 (16.5)	52 (18.3)	
≥20	661 (83.5)	232 (81.7)	
mMRC scores, (Median, IQR)	2 (1, 2)	2 (1, 3)	0.141
mMRC scores, n (%)			0.182
0–1	257 (32.4)	80 (28.2)	
≥2	535 (67.6)	204 (71.8)	
CCQ scores, (Mean ± SD)	21.2 ± 6.6	22.2 ± 6.7	**0.041**
GOLD grades, n (%)			**0.012**
1–2	478 (60.4)	147 (51.8)	
3–4	314 (39.6)	137 (48.2)	
GOLD groups, n (%)			**<0.001**
B	362 (45.7)	80 (28.2)	
E	430 (54.3)	204 (71.8)	
Comorbidities, n (%)			
Chronic heart disease	41 (5.2)	21 (7.4)	0.169
Hypertension	65 (8.2)	30 (10.6)	0.230
Lung cancer	32 (4.0)	10 (3.5)	0.698
Diabetes	13 (1.6)	6 (2.1)	0.605
Bronchiectasis	70 (8.8)	26 (9.2)	0.872
Exacerbations in the past year, (Median, IQR)	1 (0, 1.2)	1 (0, 2)	**<0.001**
Exacerbations in the past years, n (%)			**<0.001**
0	362 (45.7)	80 (28.2)	
1	232 (29.3)	92 (32.4)	
≥2	198 (25.0)	112 (39.4)	
Hospitalizations in the past year, (Median, IQR)	0 (0, 1)	0 (0, 1)	**<0.001**
Hospitalizations in the past year, n (%)			**<0.001**
0	532 (67.2)	148 (52.1)	
≥1	260 (32.8)	136 (47.9)	

Notes. The bold *p*-values indicate statistical significance.

BMI: Body Mass Index; CCQ: Clinical COPD Questionnaire; COPD: Chronic Obstructive Pulmonary Disease; CAT: COPD Assessment Test; FEV1%pred: Forced Expiratory Volume in one second percentage predicted; FEV1: Forced Expiratory Volume in one second; FVC: Forced Vital Capacity; GOLD: Global Initiative for Chronic Obstructive Lung Disease; IQR: InterQuartile Range; mMRC: modified Medical Research Council.

## Results

### Clinical characteristics of the patients

**Figure 1.**
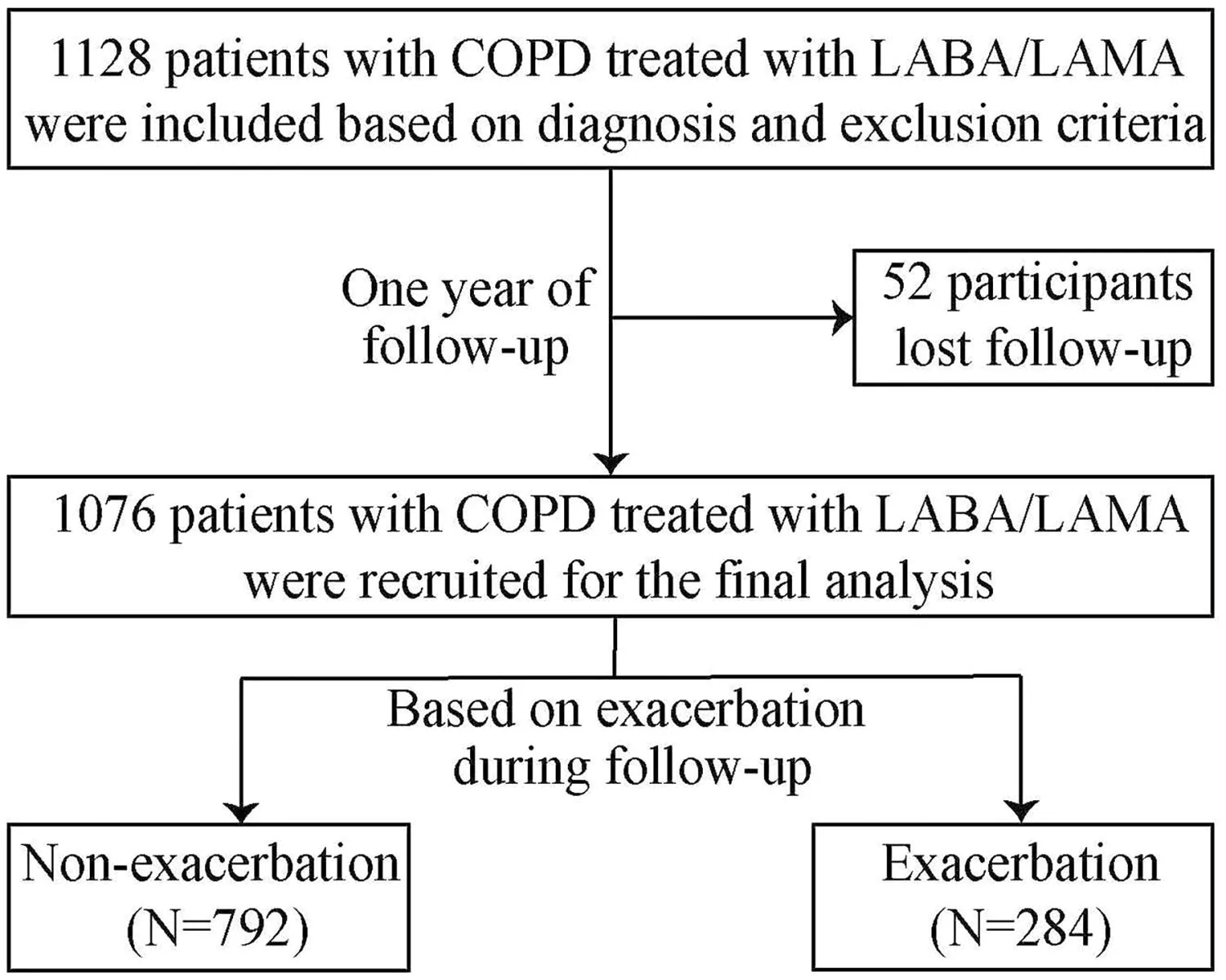
Flow chart of this study. COPD: Chronic Obstructive Pulmonary Disease; LAMA: Long-Acting Muscarinic Antagonist; LABA: Long-Acting β_2_-Agonist.

In total, 1076 patients with COPD were included in this study (Figure 1). Their mean age was 66.3 ± 8.2 years, and 88.1% of the patients were male. The mean CCQ score was 21.5 ± 5.8. The proportions of patients with GOLD grades 3–4, a history of exacerbations, and hospitalization were 41.9%, 58.9%, and 36.8%, respectively (Table 1).

During one year follow-up period, 284 (26.4%) patients experienced an exacerbation, and the median number of exacerbations was 0 (Table 2). Furthermore, there was no significant difference in future exacerbations among the different LABA/LAMA combinations used (Supplement Table 3).

### Clinical characteristics of patients experienced exacerbation during follow-up

Patients who experienced exacerbations had a higher age, and CCQ scores, and a greater number of exacerbations and hospitalizations in the past year, and a higher proportion of GOLD grades 3–4, ≥2 exacerbations in the past year, and ≥ 1 hospitalization in the past year (*p* < 0.05) (Table 3).

Multivariate logistic regression analysis showed that GOLD grades 3–4 (OR = 1.343, 95% CI = 1.016–1.776), one exacerbation in the past year (OR = 1.763, 95% CI = 1.248–2.489), and ≥2 exacerbations in the past year (OR = 2.514, 95% CI = 1.792–3.525) were independent risk factors for experiencing exacerbations during follow-up (*p* < 0.05) (Table 4).

**Table 4.** Multivariate analysis of relative factors for exacerbation in total patients.

Variables	OR	95% CI	*p*-values
GOLD grades			
1–2	Reference		
3–4	1.343	1.016–1.776	**0.038**
Exacerbations in the past years			
0	Reference		
1	1.763	1.248–2.489	**0.001**
≥2	2.514	1.792–3.525	**<0.001**

Notes. Variables in the logistic regression model including age, CCQ scores, GOLD grades, and exacerbations in the past year. The bold *p*-values indicate statistical significance.

COPD: Chronic Obstructive Pulmonary Disease; CCQ: Clinical COPD Questionnaire; GOLD: Global Initiative for Chronic Obstructive Lung Disease; OR: Odds Ratio; CI: Confidence intervals.

### Clinical characteristics of patients who experienced exacerbations during follow-up in groups B and E

In group B, multivariate logistic regression analysis showed that GOLD grades 3–4 (OR = 1.808, 95% CI = 1.096–2.981) and hypertension (OR = 3.400, 95% CI = 1.712–6.752) were independent risk factors for experiencing exacerbations during follow-up (*p* < 0.05) (Table 5).

**Table 5.** Multivariate analysis of relative factors for future exacerbations in group B patients.

Variables	Univariate			Multivariate		
	OR	95% CI	*p*-values	OR	95% CI	*p*-values
Age (years)	1.004	0.973–1.035	0.812			
Sex						
Male	Reference					
Female	1.473	0.692–3.136	0.315			
Education level						
Under junior high school	Reference					
Over high school	0.914	0.519–1.611	0.756			
BMI (kg/m^2^)	1.028	0.960–1.100	0.429			
Smoke history						
Never smoker	Reference					
Former smoker	0.737	0.377–1.440	0.372			
Current smoker	0.506	0.268–1.000	0.056			
Smoking, (pack/year)	0.999	0.991–1.001	0.777			
Biofuel exposure						
Yes	Reference					
No	0.974	0.588–1.612	0.917			
FeNO (ppb)	1.014	0.993–1.036	0.191			
Eosinophil count (×10^9^)	0.532	0.006–43.924	0.779			
Pulmonary function,						
FEV1 %pred	0.991	0.978–1.003	0.144			
FEV1/FVC	0.988	0.968–1.010	0.284			
CAT scores	1.032	0.984–1.081	0.194			
mMRC scores	1.185	0.908–1.545	0.212			
CCQ scores	1.034	0.993–1.077	0.104			
GOLD grades						
1–2	Reference			Reference		
3–4	1.657	1.018–2.700	**0.012**	1.808	1.096–2.981	**0.020**
Comorbidities						
Chronic heart disease	2.074	0.824–5.221	0.122			
Hypertension	3.102	1.581–6.084	**0.001**	3.400	1.712–6.752	**<0.001**
Lung cancer	1.413	0.448–4.453	0.555			
Diabetes	3.487	0.765–15.897	0.107			
Bronchiectasis	1.066	0.331–3.057	0.992			

Notes. Variables in the logistic regression model including GOLD grades and hypertension. The bold *p*-values indicate statistical significance.

BMI: Body Mass Index; CCQ: Clinical COPD Questionnaire; COPD: Chronic Obstructive Pulmonary Disease; CAT: COPD Assessment Test; FEV1%pred: Forced Expiratory Volume in one second percentage predicted; FEV1: Forced Expiratory Volume in one second; FVC: Forced Vital Capacity; GOLD: Global Initiative for Chronic Obstructive Lung Disease; IQR: InterQuartile Range; mMRC: modified Medical Research Council; OR: Odds Ratio; CI: Confidence intervals.

In group E, multivariate logistic regression analysis showed that ≥2 exacerbations in the past year (OR = 1.426, 95% CI = 1.021–1.993) was independent risk factors for experiencing exacerbations during follow-up (*p* < 0.05) (Table 6).

**Table 6.** Multivariate analysis of relative factors for future exacerbations in group E patients.

Variables	Univariate			Multivariate		
	OR	95% CI	*p*-values	OR	95% CI	*p*-values
Age (years)	1.020	0.999–1.041	0.057			
Sex						
Male	Reference					
Female	1.043	0.639–1.701	0.868			
Education level						
Under junior high school	Reference					
Over high school	0.811	0.542–1.214	0.309			
BMI (kg/m^2^)	0.959	0.914–1.006	0.089			
Smoke history						
Never smoker	Reference					
Former smoker	1.135	0.715–1.801	0.591			
Current smoker	0.890	0.567–1.397	0.612			
Smoking, (pack/year)	1.003	0.998–1.009	0.276			
Biofuel exposure						
Yes	Reference					
No	0.990	0.964–1.016	0.422			
FeNO (ppb)	0.989	0.993–1.036	0.191			
Eosinophil count (×10^9^)	0.904	0.526–1.552	0.714			
Pulmonary function						
FEV1 %pred	1.000	0.992–1.009	0.929			
FEV1/FVC	0.996	0.982–1.009	0.541			
CAT scores	1.009	0.982–1.036	0.527			
mMRC scores	1.062	0.903–1.250	0.468			
CCQ scores	1.005	0.981–1.029	0.705			
GOLD grades						
1–2	Reference					
3–4	1.285	0.919–1.795	0.142			
Comorbidities						
Chronic heart disease	1.194	0.607–2.348	0.608			
Hypertension	0.760	0.402–1.437	0.399			
Lung cancer	0.656	0.258–1.667	0.375			
Diabetes	0.698	0.187–2.607	0.593			
Bronchiectasis	0.879	0.518–1.491	0.632			
Exacerbations in the past years						
1	Reference			Reference		
≥2	1.426	1.021–1.993	**0.038**	1.426	1.021–1.993	**0.038**
Hospitalizations in the past year						
0	Reference					
≥1	1.308	0.922–1.855	0.132			

Notes: Variables in the logistic regression model including exacerbations in the past years. The bold *p*-values indicate statistical significance.

BMI: Body Mass Index; CCQ: Clinical COPD Questionnaire; COPD: Chronic Obstructive Pulmonary Disease; CAT: COPD Assessment Test; FEV1%pred: Forced Expiratory Volume in one second percentage predicted; FEV1: Forced Expiratory Volume in one second; FVC: Forced Vital Capacity; GOLD: Global Initiative for Chronic Obstructive Lung Disease; IQR: InterQuartile Range; mMRC: modified Medical Research Council; OR: Odds Ratio; CI: Confidence intervals.

## Discussion

As recommended in the GOLD report, dual LABA/LAMA is used as the initial inhalation therapy for patients in groups B and E [6]. Previous studies have primarily focused on comparing the efficacy of LABA/LAMA with LAMA monotherapy or LABA/ICS, and LABA/LAMA has been shown to be significantly superior [14,15]. However, in real-world clinical practice, patients remain at risk of future exacerbations despite receiving LABA/LAMA as initial inhalation therapy [10], and this issue remains insufficiently addressed in current research. In this study, we aimed to identify the risk factors for future exacerbations in patients treated with dual LABA/LAMA. The main findings demonstrated that GOLD grades 3–4 and a history of exacerbations were independent risk factors for subsequent exacerbations during follow-up. When stratified by GOLD group, GOLD grades 3–4 and hypertension predicted future exacerbations in group B, whereas ≥2 exacerbations in the past year was the key risk factor in group E. These findings highlight the heterogeneity of exacerbation risk across patient subgroups and support individualized risk stratification and management.

In the present study, nearly one-quarter of patients treated with LABA/LAMA developed exacerbations within one year, confirming that even with recommended dual bronchodilator therapy, a substantial proportion of patients remain at high risk of exacerbation. This observation is consistent with real-world evidence showing that symptom-based initial treatment may not fully prevent exacerbations in patients with advanced airflow limitation or frequent prior exacerbations [16]. Our findings underscore the need to supplement GOLD grouping with objective risk markers to identify vulnerable individuals.

Furthermore, we found that GOLD grades 3–4 was an independent risk factor for future exacerbations in the overall cohort and in patients in group B. Severe airflow limitation reflects irreversible structural damage and impaired reserve capacity, predisposing patients to rapid clinical deterioration triggered by minor insults. Lower FEV1%pred has consistently been associated with a higher exacerbation frequency, longer hospital stays, and poorer long-term prognosis [17,18]. For patients with advanced GOLD grades, intensified monitoring and early escalation to triple therapy may be warranted despite stable symptoms.

Airway inflammation constitutes the core pathogenic driver of COPD exacerbations [19]. For patients in group E, the elevated risk of exacerbations is inevitably accompanied by more severe airway inflammation. In addition, a history of exacerbations was the strongest predictor in this study, with a dose–response relationship: the risk increased progressively from one exacerbation to ≥2 exacerbations. This reinforces the concept that ‘previous exacerbation is the strongest predictor of future exacerbation’ [20]. In patients in group E, who are already defined by prior exacerbations, having ≥2 episodes in the past year further increased the risk, suggesting that these patients require early escalation to triple therapy or other preventive strategies.

Our study has several strengths. First, it reflects a real-world population, focusing on patients in groups B and E treated with first-line LABA/LAMA. Second, the large sample size and long-term follow-up improve the reliability of the findings. Third, the subgroup analyses provide actionable insights for personalized care.

Furthermore, hypertension was identified as an independent predictor only in group B, but not in group E. Pathophysiologically, this discrepancy may arise because patients in GOLD group B exhibit marked respiratory symptoms without a history of exacerbations; their increased vulnerability to subsequent exacerbations may arise predominantly from systemic complications rather than recurrent airway inflammatory episodes. Hypertension-induced endothelial dysfunction and impaired cardiopulmonary reserve may significantly increase their risk of exacerbation. By contrast, exacerbations in group E are driven predominantly by recurrent airway inflammatory episodes, and the strong predictive effect of multiple prior exacerbations may mask the systemic risk associated with hypertension in multivariate analysis [19–21]. It implied that tight blood pressure control may be particularly important for patients in GOLD group B receiving LABA/LAMA, whereas for patients in group E with two or more exacerbations in the past year, anti-inflammatory triple therapy should be prioritized regardless of hypertension status.

In addition, large pivotal trials such as IMPACT and ETHOS primarily focused on head-to-head comparisons of triple therapy, LABA/LAMA, and LAMA monotherapy, with subgroup analyses of predictive factors serving only as secondary exploratory endpoints across mixed treatment cohorts [22–24]. By contrast, our study enrolled a homogeneous cohort of patients receiving long-term LABA/LAMA maintenance therapy. Although the two core predictors were consistent with previous findings, we verified their predictive value specifically within this large intermediate-risk population independent of ICS exposure, which cannot be directly inferred from the pooled subgroup analyses of IMPACT and ETHOS. Furthermore, IMPACT and ETHOS were rigorously designed randomized controlled trials with stringent inclusion and exclusion criteria, which inevitably limited their generalizability to routine clinical practice [22–24]. Our study was based on a real-world retrospective cohort reflecting everyday prescribing patterns and patient characteristics. Reconfirming the predictive value of established exacerbation risk factors in unselected patients treated with LABA/LAMA helps bridge the evidence gap between highly controlled randomized controlled trials and routine clinical practice and strengthens the external validity of these predictors in the dual-therapy subgroup.

Several limitations should be acknowledged. This was a retrospective, single-center study, which may have introduced selection and information bias. We did not collect inflammatory biomarkers, or detailed computed tomography findings. Future prospective, multicenter studies are needed to validate these findings. In addition, excluding high-risk patients who required escalation to triple therapy limits the generalizability of the model to this subgroup, and dedicated studies focusing on patients receiving triple therapy cohort can be carried out in the future. Furthermore, this was a real-world observational study, we did not assess inhaler technique or adherence during data collection. We also acknowledge that the exact timing of individual exacerbations was not available from the telephone follow-up data, which represents another limitation of the study. Finally, data on influenza, pneumococcal, and COVID-19 vaccination during the follow-up period were not available, which may have influenced exacerbation risk.

## Conclusions

Patients with COPD treated with LABA/LAMA remained at high risk of future exacerbations. GOLD grades 3–4 and exacerbations in the past year were associated with future exacerbations. Clinicians may integrate pulmonary function and exacerbation history into risk stratification. Early intervention and tailored therapy may help reduce exacerbations and improve clinical outcomes.

## Supplementary Material

Supplement tables.docx

## Data Availability

All data of this study are available from the corresponding author for reasonable request.

## References

[1] Chen S, Kuhn M, Prettner K, et al. The global economic burden of chronic obstructive pulmonary disease for 204 countries and territories in 2020-50: a health-augmented macroeconomic modelling study. Lancet Glob Health. 2023;11(8):e1183–e1193. doi: 10.1016/S2214-109X(23)00217-6. 37474226 PMC10369014

[2] Yin P, Wu J, Wang L, et al. The burden of COPD in China and its provinces: findings from the global burden of disease study 2019. Front Public Health. 2022;10:859499. doi: 10.3389/fpubh.2022.859499. 35757649 PMC9215345

[3] Donaldson GC, Seemungal TA, Bhowmik A, et al. Relationship between exacerbation frequency and lung function decline in chronic obstructive pulmonary disease. Thorax. 2002;57(10):847–852. doi: 10.1136/thorax.57.10.847. 12324669 PMC1746193

[4] Ruan H, Zhang H, Wang J, et al. Readmission rate for acute exacerbation of chronic obstructive pulmonary disease: a systematic review and meta-analysis. Respir Med. 2023;206:107090. doi: 10.1016/j.rmed.2022.107090. 36528962

[5] Sethi S, Make BJ, Robinson SB, et al. Relationship of COPD exacerbation severity and frequency on risks for future events and economic burden in the medicare fee-for-service population. Int J Chron Obstruct Pulmon Dis. 2022;17:593–608. doi: 10.2147/COPD.S350248. 35342290 PMC8948172

[6] GOLD Executive Committee. Global strategy for the diagnosis, management and prevention of chronic obstructive pulmonary disease. REPORT; 2026. [cited November, 2025]. Available from https://goldcopd.org/

[7] Fukuda N, Horita N, Kaneko A, et al. Long-acting muscarinic antagonist (LAMA) plus long-acting beta-agonist (LABA) versus LABA plus inhaled corticosteroid (ICS) for stable chronic obstructive pulmonary disease. Cochrane Database Syst Rev. 2023;6(6):CD012066. doi: 10.1002/14651858.CD012066.pub3. 37276335 PMC10241721

[8] Monteagudo M, Nuñez A, Barrecheguren M, et al. Effectiveness of Treatment With Dual Bronchodilation (LABA/LAMA) compared with combination therapy (LABA/ICS) for patients with COPD: a population-based study. Arch Bronconeumol. 2022;58(10):699–707. doi: 10.1016/j.arbres.2022.05.002. 35618580

[9] Suissa S, Cherian M, Dell’Aniello S, et al. Initial treatment of COPD with LABA-LAMA versus LAMA: real-world comparative effectiveness and safety. COPD. 2026;23(1):2622739. doi: 10.1080/15412555.2026.2622739. 41709817

[10] Song Q, Cheng W, Liu C, et al. The future exacerbation and mortality of different inhalation therapies among patients with chronic obstructive pulmonary disease in various GOLD groups: a focus on the GOLD 2017 and GOLD 2023 reports. Ther Adv Respir Dis. 2023;17:17534666231213715. doi: 10.1177/17534666231213715. 38018090 PMC10685753

[11] Cao J, Sun T, Yang H, et al. The risk factors of future exacerbations and treatment responses among different inhalation therapies of patients with preserved ratio impaired spirometry. J Glob Health. 2026;16:04070. doi: 10.7189/jogh.16.04070. 41717986 PMC12922469

[12] Duan J-X, Cheng W, Zeng Y-Q, et al. Characteristics of patients with chronic obstructive pulmonary disease exposed to different environmental risk factors: a large cross-sectional study. Int J Chron Obstruct Pulmon Dis. 2020;15:2857–2867. doi: 10.2147/COPD.S267114. 33192059 PMC7654530

[13] Liu C, Cheng W, Zeng Y, et al. Different characteristics of ex-smokers and current smokers with COPD: a cross-sectional study in China. Int J Chron Obstruct Pulmon Dis. 2020;15:1613–1619. doi: 10.2147/COPD.S255028. 32753861 PMC7354950

[14] Rodrigo GJ, Price D, Anzueto A, et al. LABA/LAMA combinations versus LAMA monotherapy or LABA/ICS in COPD: a systematic review and meta-analysis. Int J Chron Obstruct Pulmon Dis. 2017;12:907–922. doi: 10.2147/COPD.S130482. 28360514 PMC5364009

[15] Chen C-Y, Chen W-C, Huang C-H, et al. LABA/LAMA fixed-dose combinations versus LAMA monotherapy in the prevention of COPD exacerbations: a systematic review and meta-analysis. Ther Adv Respir Dis. 2020;14:1753466620937194. doi: 10.1177/1753466620937194. 32643547 PMC7350046

[16] Song Q, Lin L, Li T, et al. The treatment responses among different inhalation therapies for GOLD group E patients with chronic obstructive pulmonary disease. J Glob Health. 2025;15:04055. doi: 10.7189/jogh.15.04055. 39977672 PMC11842004

[17] Almagro P, Martinez-Camblor P, Soriano JB, et al. Finding the best thresholds of FEV1 and dyspnea to predict 5-year survival in COPD patients: the COCOMICS study. PLoS One. 2014;9(2):e89866. doi: 10.1371/journal.pone.0089866. 24587085 PMC3937394

[18] Ke X, Marvel J, Yu T-C, et al. Impact of lung function on exacerbations, health care utilization, and costs among patients with COPD. Int J Chron Obstruct Pulmon Dis. 2016;11:1689–1703. doi: 10.2147/COPD.S108967. 27555759 PMC4968671

[19] Tumkaya M, Atis S, Ozge C, et al. Relationship between airway colonization, inflammation and exacerbation frequency in COPD. Respir Med. 2007;101(4):729–737. doi: 10.1016/j.rmed.2006.08.020. 17002892

[20] Hurst JR, Vestbo J, Anzueto A, et al. Susceptibility to exacerbation in chronic obstructive pulmonary disease. N Engl J Med. 2010;363(12):1128–1138. doi: 10.1056/NEJMoa0909883. 20843247

[21] Wedzicha JA, Brill SE, Allinson JP, et al. Mechanisms and impact of the frequent exacerbator phenotype in chronic obstructive pulmonary disease. BMC Med. 2013;11(1):181. doi: 10.1186/1741-7015-11-181. 23945277 PMC3750926

[22] Stolz D, Hermansson E, Ouwens M, et al. Mortality risk reduction with budesonide/glycopyrrolate/formoterol fumarate versus fluticasone furoate/umeclidinium/vilanterol in COPD: a matching-adjusted indirect comparison based on ETHOS and IMPACT. Curr Med Res Opin. 2023;39(10):1395–1405. doi: 10.1080/03007995.2023.2247969. 37583267

[23] Rabe KF, Martinez FJ, Ferguson GT, et al. Triple inhaled therapy at two glucocorticoid doses in moderate-to-very-severe COPD. N Engl J Med. 2020;383(1):35–48. doi: 10.1056/NEJMoa1916046. 32579807

[24] Lipson DA, Barnhart F, Brealey N, et al. Once-daily single-inhaler triple versus dual therapy in patients with COPD. N Engl J Med. 2018;378(18):1671–1680. doi: 10.1056/NEJMoa1713901. 29668352

