# Supplementary material for: Risk factors for future exacerbations in patients with COPD treated with dual LABA/LAMA
Source: Ann Med. 2026 Sep 15;58(1):2722722. doi: 10.1080/07853890.2026.2722722 (PMC13580378; doi:10.1080/07853890.2026.2722722)
Supplement: Supplementary file 1 [file IANN_A_2722722_SM2694.docx]

**Supplement table 1. The results of collinearity test among the variables in the logistic regression model in total patients.**

| **Variables** | **Tolerance** | **VIF** |
| --- | --- | --- |
| Age | 0.980 | 1.021 |
| CCQ | 0.890 | 1.124 |
| Exacerbations in the past year | 0.963 | 1.038 |
| GOLD grades | 0.933 | 1.072 |

**Abbreviations:** COPD, Chronic Obstructive Pulmonary Disease; CCQ, Clinical COPD Questionnaire; GOLD, Global Initiative for Chronic Obstructive Lung Disease; VIF, [Variance Inflation Factor](https://www.so.com/link?m=eRnXGmFyaWnASugeVE4o6xNexy0c9u8sDpZJ/lyVuTIsHgPO6DCY/QsXdXiDclRqjooIwN8x6KFfzvkfEhygnScG66MwdnDS+LDVR8w7mCM9vYLMwZKeYzKEWBpfYy6g6AwYrCaVEATxoesNPqX64DEcSgzA1w36w0kl6W5otBgg6W9iJ3+h1jqOxJ54egGgkVq4LJA==).

**Supplement table 2. The results of collinearity test among the variables in the logistic regression model in group B patients.**

| **Variables** | **Tolerance** | **VIF** |
| --- | --- | --- |
| Hypertension | 0.95 | 1.005 |
| GOLD grades | 0.995 | 1.005 |

**Abbreviations:** GOLD, Global Initiative for Chronic Obstructive Lung Disease; VIF, [Variance Inflation Factor](https://www.so.com/link?m=eRnXGmFyaWnASugeVE4o6xNexy0c9u8sDpZJ/lyVuTIsHgPO6DCY/QsXdXiDclRqjooIwN8x6KFfzvkfEhygnScG66MwdnDS+LDVR8w7mCM9vYLMwZKeYzKEWBpfYy6g6AwYrCaVEATxoesNPqX64DEcSgzA1w36w0kl6W5otBgg6W9iJ3+h1jqOxJ54egGgkVq4LJA==).

**Supplement table 3. The future exacerbation of the patients with the specific LABA/LAMA combinations used during one year of follow-up.**

| **Variables** | **Glycopyrronium Bromide/Formoterol Fumarate**  **(n=201)** | **Indacaterol Maleate/Glycopyrronium Bromide**  **(n=299)** | **Umeclidinium Bromide/Vilanterol Trifenatate**  **(n=576)** | **P -**  **values** |
| --- | --- | --- | --- | --- |
| Exacerbations during one year, (Median, IQR) | 0 (0, 1) | 0 (0, 1) | 0 (0, 0) | 0.221 |
| Exacerbations, n (%) |  |  |  | 0.346 |
| Yes | 55 (27.4) | 87 (29.1) | 142 (24.7) |  |
| No | 146 (72.6) | 212 (70.9) | 434 (75.3) |  |

**Abbreviations:** IQR, InterQuartile Range; LAMA, Long-Acting Muscarinic Antagonist; LABA, Long-Acting β2-Agonist.
